# Rice Stripe Virus Coat Protein-Mediated Virus Resistance Is Associated With RNA Silencing in *Arabidopsis*

**DOI:** 10.3389/fmicb.2020.591619

**Published:** 2020-11-13

**Authors:** Feng Sun, Peng Hu, Wei Wang, Ying Lan, Linlin Du, Yijun Zhou, Tong Zhou

**Affiliations:** ^1^Jiangsu Key Laboratory for Food Quality and Safety-State Key Laboratory Cultivation Base of Ministry of Science and Technology, Institute of Plant Protection, Jiangsu Academy of Agricultural Sciences, Nanjing, China; ^2^The State Key Laboratory of Crop Genetics and Germplasm Enhancement, Nanjing Agricultural University, Nanjing, China; ^3^College of Plant Protection, Nanjing Agricultural University, Nanjing, China

**Keywords:** Rice stripe virus, CP-mediated resistance, RNA silencing, deep sequence, *dcl2/3/4*

## Abstract

Rice stripe virus (RSV) causes rice stripe disease, which is one of the most serious rice diseases in eastern Asian countries. It has been shown that overexpression of RSV coat protein (CP) in rice plants enhances resistance against virus infection. However, the detailed mechanism underlying RSV CP-mediated virus resistance remains to be determined. In this study, we show that both translatable and non-translatable RSV CP transgenic *Arabidopsis* plants exhibited immunity to virus infection. By using deep sequencing analysis, transgene-derived small interfering RNAs (t-siRNAs) from non-translatable CP transgenic plants and virus-derived small interfering RNAs (vsiRNAs) mapping in the CP region from RSV-infected wild-type plants showed similar sequence distribution patterns, except for a significant increase in the abundance of t-siRNA reads compared with that of CP-derived vsiRNAs. To further test the correlation of t-siRNAs with RSV immunity, we developed RSV CP transgenic *Arabidopsis* plants in an siRNA-deficient *dcl2/3/4* mutant background, and these CP transgenic plants showed the same sensitivity to RSV infection as non-transgenic plants. Together, our data indicate that the expression of RSV CP protein from a transgene is not a prerequisite for virus resistance and RSV CP-mediated resistance is mostly associated with the RNA silencing mechanism in *Arabidopsis* plants.

## Introduction

RNA silencing is a conserved antiviral defense mechanism that has been used to increase resistance to plant virus infections (Pumplin and Voinnet, 2013; Guo et al., 2019). In antiviral RNA silencing, host Dicer-like ribonucleases (DCLs) cleave viral double-stranded RNAs (dsRNAs) that are formed during virus replication and transcription into 21-24-nucleotide (nt) viral small interfering RNAs (vsiRNAs) (Pumplin and Voinnet, 2013; Guo et al., 2019). Secondary vsiRNAs can be produced by dicing secondary viral dsRNA synthesized by host-encoded RNA-dependent RNA polymerases (RdRps) (Wassenegger and Krczal, 2006; Zhang et al., 2015). These vsiRNAs are mainly loaded into the Argonaute (Ago) protein-containing RNA-induced silencing complex (RISC) to posttranscriptionally repress target viral and host RNAs by sequence complementarity (Carbonell and Carrington, 2015; Fang and Qi, 2016). The *Arabidopsis thaliana* genome encodes four DCLs, among which DCL1 produces microRNA, whereas DCL2, DCL3, and DCL4 produces 22-, 24-, and 21-nt vsiRNAs, respectively (Guo et al., 2019). DCL4 and DCL2 are the primary components of antiviral defense in plants and exhibit redundant or cooperative functions (Diaz-Pendon et al., 2007; Guo et al., 2019). The 21-nt vsiRNA produced by DCL4 is the most dominant species of vsiRNA in RNA virus-infected plants (Deleris et al., 2006; Wang et al., 2010). DCL2-dependent 22-nt vsiRNAs also accumulate abundantly when DCL4 is absent or suppressed by viruses, and these 22-nt vsiRNAs seem to be less effective at mediating antiviral RNA silencing (Wang et al., 2011). DCL3 produces 24-nt siRNAs that are associated with transcriptional repression of transposons and repeat sequences in plants (Blevins et al., 2015; Singh et al., 2019) and may enhance antiviral defense against DNA viruses (Raja et al., 2008; Jackel et al., 2016). In *Arabidopsis* plants, AGO1 and AGO2 are the two major plant antiviral argonautes against RNA viruses by associating with vsiRNAs based on the identity of the 5′ terminal nucleotide (Mi et al., 2008; Carbonell and Carrington, 2015).

Rice stripe virus (RSV) is the type species in the genus *Tenuivirus* and causes rice stripe disease, which is one of the most serious rice diseases in eastern Asia (Falk and Tsai, 1998; Xiong et al., 2008). It is transmitted by the small brown planthopper (SBPH) (*Laodelphax striatellus* Fallén) in a circulative and propagative manner (Falk and Tsai, 1998; Li et al., 2015). Under experimental control, RSV also infects *Nicotiana benthamiana* by mechanical sap inoculation and *Arabidopsis* plants by viruliferous SBPH inoculation (Xiong et al., 2008; Sun et al., 2011, 2016). The genome of RSV contains four single-stranded RNA segments, RNA1, RNA2, RNA3, and RNA4, in order of their decreasing molecular weights (Toriyama and Watanabe, 1989). RNA1 is negative sense and encodes a putative viral RdRp (Barbier et al., 1992). The other small three RNA segments encode two proteins using an ambisense coding strategy. RNA2 encodes NS2, a viral RNA silencing suppressor (Du et al., 2011), and NSvc2, a putative membrane glycoprotein (Yao et al., 2014). RNA3 encodes NS3, another RNA silencing suppressor (Xiong et al., 2009) and a coat protein (CP). SP (a disease-specific protein) (Kong et al., 2014) and NSvc4 (a movement protein) (Xiong et al., 2008) are encoded by RNA4.

To manage rice stripe disease caused by RSV, the use of insecticides against SBPH vectors and the exploitation of genetically resistant cultivars are the most effective approaches; however, they all have concerning features, such as high costs, environmental pollution, insecticide resistance and limited resistance resources (Otuka, 2013). Recently, RNA silencing-based resistance has been shown to be an effective and powerful method to enhance plant resistance against viruses in transgenic plants (Galvez et al., 2014; Lindbo and Falk, 2017). Plants transformed with a hairpin construct consisting of an inverted repeat virus-specific sequence are able to induce RNA silencing and exhibit immunity to virus infection (Lindbo and Falk, 2017). For RSV, transgenic rice plants with the RSV CP or NSvc4 hairpin constructs exhibit near immunity against RSV infection (Shimizu et al., 2011; Sasaya et al., 2014). Chimeric CP/SP RNA silencing construct transgenic rice plants show strong resistance against two different RSV isolates (Ma et al., 2011). In addition to hairpin constructs, plant expression of the viral CP gene also confers plant viral resistance, which is mostly but not completely due to an RNA silencing-mediated resistance mechanism (Lindbo and Falk, 2017). It has been shown that overexpression of the RSV CP gene in transgenic rice plants enhances resistance against the virus (Hayakawa et al., 1992). Recent research has also shown that RSV CP mediates virus resistance through jasmonate-AGO18 signaling in rice plants (Han et al., 2020; Yang et al., 2020). However, considering that AGO18 is the key component of RNA silencing in rice plants, whether RSV CP-mediated virus resistance involves the RNA silencing pathway remains to be addressed.

In this study, using the RSV-*Arabidopsis* pathosystem, we showed that both translatable and non-translatable RSV CP transgenic *Arabidopsis* plants exhibit immunity to RSV infection. By using deep sequencing analysis and comparing transgenic-derived siRNAs (t-siRNAs) from non-translatable versions of CP transgenic plants and CP-derived virus-derived siRNAs (vsiRNAs) from RSV-infected wild-type plants, we found that siRNA mapped to the CP sequence segment showed a similar pattern in both transgenic plants and virus-infected wild-type plants, except that greater accumulation of siRNAs was seen in transgenic immune plants. In the siRNA-deficient *dcl2/3/4* mutant background, CP transgenic plants showed the same RSV susceptibility as non-transgenic plants. Together, our collective results indicated that RSV CP-mediated virus resistance depends on the function of DCLs and mostly involved in RNA silencing in *Arabidopsis* plants.

## Materials and Methods

### Sources of Virus, Vectors, and Plant Materials

Rice stripe virus-infected rice plants were collected from Jiangsu Province in China, and the virus was confirmed by using a western blot assay (Sun et al., 2016). Non-viruliferous instar nymphs of SBPHs reared on rice seedlings (*Oryza sativa* L. cv. Wuyujing No. 3) were collected and fed on RSV-infected rice plants for 3 days to acquire the virus. The virus was maintained by successive transovarial infections in SBPHs on rice seedlings in an insect-rearing room at 25°C.

*Arabidopsis thaliana* (Col-0) and *dcl2/3/4* mutant seeds were obtained from Dr. Xiuren Zhang (Texas A&M University, College Station, United States) and grown in potting soil in a growth chamber at 23°C under 200 μmol m^–2^ s^–1^ illumination and 16-h light/8-h dark cycle conditions.

### RSV Inoculation Assay

Transgenic and wild-type *Arabidopsis* plants at 2 weeks were inoculated with 10 viruliferous SBPHs per plant and were kept in a glass incubator containing ten plants. SBPHs were sprayed with insecticide after a 4-day inoculation access period. Plants were kept in a growth chamber for symptom development, and non-viruliferous insects were used for mock inoculation. To evaluate of resistance to RSV, thirty *Arabidopsis* plants of each line were inoculated with RSV. The seedlings were harvested at different time points for RNA and protein extractions. Each RSV inoculation assay was repeated three times.

### DNA Constructs and Transgenic Plants

The RSV *CP* gene was amplified from RSV-infected rice plant leaf tissue using RT-PCR performed with CP-specific primers (Supplementary Table S1). The CP PCR products were cloned into the pDONR-zero vector (Invitrogen, Carlsbad, CA, United States) and subsequently transferred into the binary vector pBA-Flag-Myc4-DC (Zhang et al., 2005) using the Gateway cloning system following the manufacturer’s instructions (Invitrogen Corporation). The constructed binary vector pBA-Flag-Myc4-CP was transformed into *Arabidopsis* plants using *Agrobacterium tumefaciens* strain ABI by the floral dip method (Clough and Bent, 1998; Zhang et al., 2006). The CP overexpression transgenic *Arabidopsis* plants were selected on standard MS medium containing 10 mg/L glufosinate ammonium (Sigma-Aldrich, St. Louis, MO, United States).

### Western Blot Assay

Western blot was performed following the protocol described by Sun et al. (2016). In brief, *Arabidopsis* total proteins were separated by 10% SDS-PAGE and transferred to PVDF membranes. The membranes were blocked and inoculated with an anti-RSV SP (our laboratory), anti-Myc (Sigma-Aldrich, St. Louis, MO, United States), anti-YFP (Genscript, Nanjing, China) or anti-actin antibody (Enogene, Nanjing, China) overnight at 4°C. Signals were developed in ECL buffer (Transgen Biotech, Beijing, China) and recorded with a Tanon 5200 Luminescent Imaging Workstation (Tanon, Shanghai, China).

### RT-PCR

Total RNA was isolated from the plant samples using RNAiso Plus reagent (Takara, Dalian, China), and first-strand cDNA was synthesized from 1 μg of total RNA using an iScript^TM^ cDNA Synthesis Kit (Bio-Rad, Hercules, CA, United States). PCR amplification was performed using 2 × Taq Master Mix (Vazyme Biotech, Nanjing, China) with RSV CP-specific primers (Supplementary Table S1). *EF1a* was used as a loading control.

### Quantitative Reverse-Transcription PCR

Total RNA was isolated from the plant samples using RNAiso Plus reagent (Takara, Dalian, China), and cDNA synthesis and PCR were performed as described previously (Sun et al., 2016). Briefly, first-strand cDNA was synthesized from 1 μg of total RNA using an iScript^TM^ cDNA Synthesis Kit (Bio-Rad, Hercules, CA, United States). qRT-PCR was performed using SsoFast EvaGreen Supermix (Bio-Rad, Hercules, CA, United States) with a Bio-Rad iQ5 Real-Time PCR system with gene-specific primers (Supplementary Table S1). *EF1a* was used as an internal standard, and all qRT-PCR experiments were performed at least three times.

### Deep Sequencing and Analysis of Small RNA Sequences

Total RNA was extracted from RSV-infected and mock-treated NOP-CP transgenic or non-transgenic plants each with triplicate biological replicates, using RNAiso Plus reagent (Takara, Dalian, China), and used for small RNA library construction. A total of 12 small RNA libraries were sequenced using an Illumina HiSeq 4000 sequencer to generate single-end 50-bp sequences at the Beijing Genome Institute (BGI, Shenzhen, China). Raw data were obtained after removing the adapter sequences using cutadapt (v1.16) (Martin, 2011). The clean data were then mapped to the RSV genome (NCBI accession numbers: NC_003755.1, NC_003754.1, NC_003776.1, NC_003753.1), allowing up to one mismatch. Reads showing matches with the RSV reference sequences were retained and further analyzed using the CLC Genomics Workbench program (QIAGEN, Hilden, Germany), Perl scripts and Excel tools. Small RNA-seq data was deposited in the NCBI BioProject database with accession code PRJNA649376.

## Results

### RSV CP Transgenic *Arabidopsis* Plants Show Immunity to RSV Infection

**FIGURE 1:**
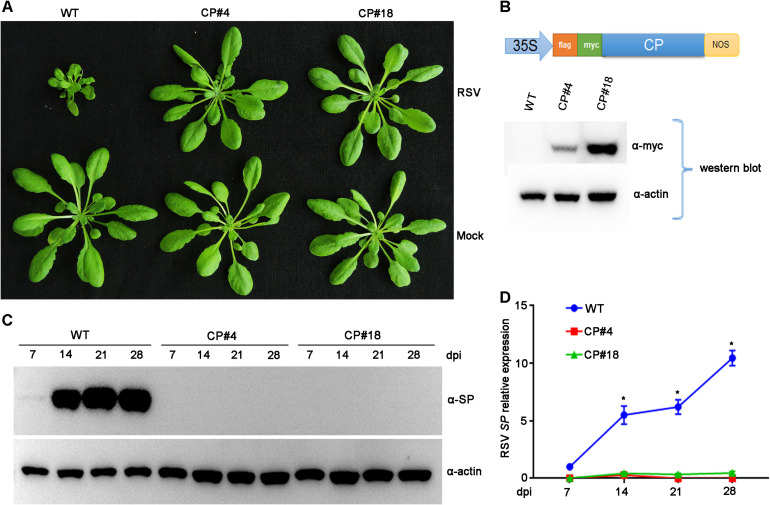
Examination of the Rice stripe virus (RSV) resistance of CP transgenic *Arabidopsis* plants. **(A)** Symptoms of mock-inoculated and RSV-infected CP transgenic (CP#4, CP#18) and wild-type (WT) *Arabidopsis* plants. Photographs were taken at 4 weeks post-inoculation. **(B)** The upper panel shows the schematic representation of the constructed binary vector pBA-Flag-Myc4-CP, and the lower panel shows western blot analysis of CP transgenic lines with an anti-Myc antibody. The actin protein level served as a loading control. **(C)** Western blot analysis of the time course of RSV-encoded SP protein accumulation in RSV-infected CP transgenic (CP#4, CP#18) and WT *Arabidopsis* plants using an SP-specific antibody. The actin protein level served as a loading control. **(D)** qRT-PCR analysis of the time course of RSV SP mRNA transcription levels in RSV-infected CP transgenic and WT *Arabidopsis* plants. Signal intensities for each transcript were normalized with those for *EF1-α*. Values are means ± SD (*n* = 3). *p ≤ 0.05 (Student’s *t*-test).

Previous studies demonstrated that transgenic rice plants expressing RSV CP exhibited a significant level of resistance to virus infection (Hayakawa et al., 1992; Yang et al., 2020). Our previous studies also showed that RSV infected *Arabidopsis* plants and caused significant symptoms, including stunted growth and vein chlorosis on leaves (Sun et al., 2011, 2016). To test whether RSV CP-mediated resistance can be extended to *Arabidopsis* plants, we first generated CP transgenic *Arabidopsis* plants. Full-length RSV CP was cloned from virus-infected rice plants and transferred into the binary vector pBA-Flag-Myc4-DC (Zhang et al., 2005) using the Gateway cloning system (Figure 1B). This recombinant vector was transformed into *Arabidopsis* plants (Col-0 ecotype) by the floral dip method (Clough and Bent, 1998; Zhang et al., 2006). These T2 generation transgenic plants overexpressing RSV CP with Flag-Myc4 epitopes were examined by western blot assay. The expression of Flag-Myc4-CP was detected only in transgenic plants with an anti-Myc antibody (Figure 1B).

To test RSV resistance, both CP transgenic (CP#4, CP#18) and wild-type (Col-0 ecotype) *Arabidopsis* plants were challenged with RSV by viruliferous SBPHs at 2-week-old stages. In three independent experiments, all wild-type plants showed typical RSV symptoms, including severe stunting at 4 weeks post-inoculation. In contrast, all the CP transgenic *Arabidopsis* plants did not show any symptoms and displayed resistance to RSV infection (Figure 1A). Moreover, RSV-encoded SP protein was detected in wild-type *Arabidopsis* plants by western blot assay at 14 to 28 days post-inoculation (dpi) but not in CP transgenic plants (Figure 1C). qRT-PCR assays showed that RSV SP mRNA accumulated to significantly higher levels in wild-type plants than in CP transgenic plants (Figure 1D). These results therefore show that RSV CP induces virus resistance in *Arabidopsis* plants.

### Expression of CP Protein From a Transgene Is Not a Prerequisite for Resistance in *Arabidopsis* Plants

**FIGURE 2:**
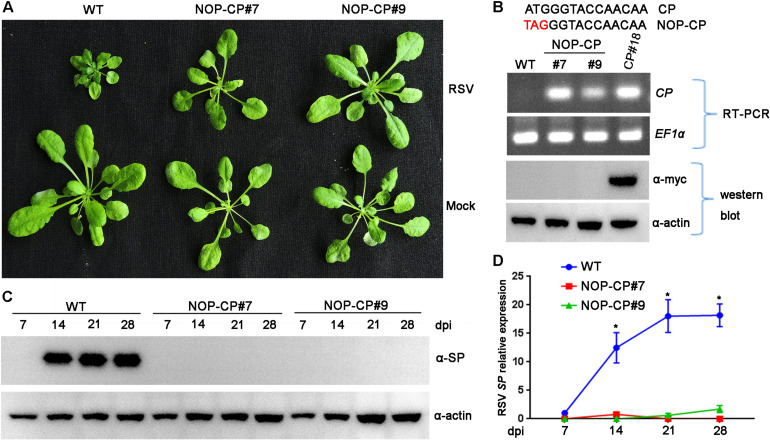
Evaluation of NOP-CP transgenic *Arabidopsis* plants for resistance to Rice stripe virus (RSV). **(A)** Symptoms of mock-inoculated and RSV-infected NOP-CP transgenic (NOP-CP#7, NOP-CP#9) and wild-type (WT) *Arabidopsis* plants. Photographs were taken at 3 weeks post-inoculation. **(B)** The upper panel shows the schematic representation of the NOP-CP nucleotide sequence. The middle panel shows RT-PCR analysis of NOP-CP mRNA transcription levels of NOP-CP transgenic lines, and the EF1-α mRNA level served as a loading control. The lower panel shows western blot analysis of NOP-CP transgenic lines with an anti-Myc antibody, and the actin protein level served as a loading control. **(C)** Western blot analysis of the time course of RSV-encoded SP protein accumulation in RSV-infected NOP-CP transgenic (NOP-CP#7, NOP-CP#9) and WT *Arabidopsis* plants using an SP-specific antibody. The actin protein level served as a loading control. **(D)** qRT-PCR analysis of the time course of RSV SP mRNA transcription levels in RSV-infected NOP-CP transgenic and Col-0 *Arabidopsis* plants. Signal intensities for each transcript were normalized with those for *EF1-α*. Values are means ± SD (*n* = 3). *p ≤ 0.05 (Student’s *t*-test).

Previous studies demonstrated that the mechanism of CP-mediated resistance depended on the particular virus system and transgenic plants being analyzed (Lindbo and Falk, 2017). To demonstrate whether expression of RSV CP protein is necessary for resistance, we generated *Arabidopsis* plants overexpressing a non-translatable protein mutant of RSV CP (NOP-CP) in which the first codon ATG of the CP gene was replaced with TAG (Figure 2B), and the NOP-CP sequence was inserted into the pBA-Flag-Myc4-DC vector and tagged with Flag-Myc4 epitopes. These T2 generation transgenic plants were confirmed by RT-PCR and western blot assays. As expected, transcripts of NOP-CP mRNA were detected with primers specific to the CP sequence only in transgenic *Arabidopsis* plants (Figure 2B). However, signals of NOP-CP protein were not visible in either transgenic or wild-type plants with an anti-Myc antibody. These results indicate that NOP-CP transgenic *Arabidopsis* plants expressed only CP mRNA but not protein. To further analyze whether there was still a truncated RSV CP protein encoding C-terminal truncation in the corresponding region of CP nucleotides in NOP-CP gene. YFP-tagged CP or NOP-CP in C-terminal were transiently expressed in *Nicotiana benthamiana*. The transcripts of NOP-CP-YFP mRNA were detected in *Nicotiana benthamiana* (Supplementary Figure S1B). However, signals of NOP-CP-YFP protein were not visible in tobacco plants using confocal imaging and western blot assays (Supplementary Figures S1A,C). These data indicated that NOP-CP-YFP gene expressed only CP mRNA but not protein in *Nicotiana benthamiana*.

To test RSV resistance, NOP-CP transgenic (NOP-CP#7, NOP-CP#9) and wild-type *Arabidopsis* plants were inoculated with RSV viruliferous SBPHs. In three independent trials, no RSV symptoms were observed in the NOP-CP transgenic *Arabidopsis* plants (Figure 2A), in contrast with the severe stunting symptoms in wild-type plants. Consistently, the accumulation of viral SP protein was not detected in the NOP-CP transgenic plants by western blot (Figure 2C), and fewer SP mRNA transcripts were detected in NOP-CP transgenic plants than in wild-type plants (Figure 2D). These results show that *Arabidopsis* transgenic plants with a non-translatable RSV CP sequence are immune to virus infection and that RSV CP-mediated resistance is independent of CP protein expression.

### Deep Sequencing of Small RNA From NOP-CP Transgenic and Wild-Type *Arabidopsis* Plants

**FIGURE 3:**
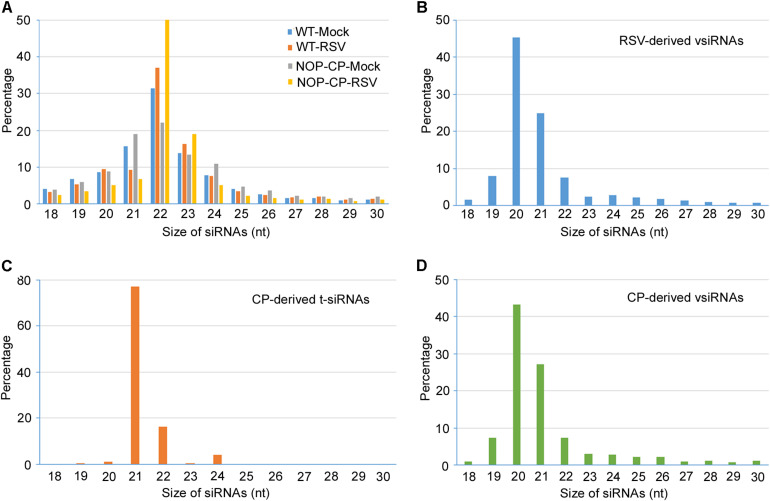
Size distribution of 18–30 nucleotide small RNAs. **(A)** Size distribution of total small RNA from mock-inoculated and RSV-infected NOP-CP transgenic and Col-0 *Arabidopsis* plants. **(B)** Size distribution of small RNA from vsiRNAs from RSV-infected Col-0 *Arabidopsis* plants. **(C)** Size distribution of CP-derived t-siRNAs from NOP-CP transgenic plants. **(D)** Size distribution of CP-derived vsiRNAs from RSV-infected Col-0 plants.

To better resolve whether the small RNA population is associated with RSV CP-mediated resistance and minimize the impact of CP protein on the immune response, 12 libraries were constructed with small RNA isolated from mock- and RSV-infected NOP-CP transgenic and wild-type *Arabidopsis* plants. Each library generated 7–15 million clean reads ranging from 18 to 30 nt (Table 1). Three biological replicates of each group were analyzed and showed very similar patterns. The results described below were derived from the average generated from three biological replicates. The size distributions of total small RNAs within these different treatments were similar; the dominant size was 22 nt, followed by 21 nt and 23 nt (Figure 3A). We mapped clean small RNA (18–30 nt) reads to the RSV genome and CP transgenic sequence and obtained RSV-derived, CP-derived siRNAs. In RSV-infected wild-type *Arabidopsis* plants, we identified 160,436 vsiRNAs, accounting for 1.55% of the total, whereas only 1,481 reads matched to the RSV genome in mock control plants. In NOP-CP transgenic *Arabidopsis* plants, almost all reads matched to the RSV genome were generated from the NOP-CP segment sequence, accounting for 0.09% of the total reads in RSV-infected plants and 0.27% in mock plants (Table 1). Unexpectedly, in NOP-CP transgenic *Arabidopsis* plants, RSV infection resulted in less accumulation of NOP-CP-derived small RNA than in mock plants despite generating more small RNA reads mapped to other RSV genomic regions (Table 1). The sequencing results confirmed that the accumulation of NOP-CP-derived siRNAs from transgenic sequences was associated with the immunity of these plants to RSV infection.

**TABLE 1:** Twelve libraries constructed with small RNAs from wild-type (WT) and NOP-CP transgenic (NOP-CP) *Arabidopsis* plants after mock control (Mock) or RSV inoculation (RSV), including three biological replicates each.

Library	Total 18–30 nt reads	Number of reads on RSV	Percentage of RSV reads (%)	Number of reads on CP	Percentage of CP reads (%)
NOP-CP-Mock1	8,955,649	20,391	0.23	20,326	0.23
NOP-CP-Mock2	7,625,242	13,531	0.18	13,455	0.18
NOP-CP-Mock3	9,991,991	40,589	0.41	40,473	0.41
NOP-CP-Mock (average)	8,857,627	24837	0.27	24751	0.27
NOP-CP-RSV1	11,577,160	9,423	0.08	8,058	0.07
NOP-CP-RSV2	8,858,390	9,017	0.10	7,610	0.09
NOP-CP-RSV3	9,430,632	7,036	0.07	6,826	0.07
NOP-CP-RSV (average)	9,955,394	8492	0.09	7498	0.08
WT-Mock1	14,727,568	64	0.00	1	0.00
WT-Mock2	15,054,977	71	0.00	0	0.00
WT-Mock3	15,943,020	4,307	0.03	333	0.00
WT-Mock (average)	15,241,855	1,481	0.01	111	0.00
WT-RSV1	8,726,871	69,622	0.80	4,878	0.06
WT-RSV2	9,272,442	111,136	1.20	10,252	0.11
WT-RSV3	11,340,961	300,550	2.65	23,138	0.20
WT-RSV (average)	9,780,091	160436	1.55	12756	0.12

### Characterization of vsiRNA in RSV-Infected Wild-Type *Arabidopsis* Plants

To further decipher the regulatory functions of vsiRNAs, we studied the profiles of vsiRNA in RSV-infected *Arabidopsis* plants. Size distribution analysis showed that RSV-derived vsiRNAs were predominantly 20 and 21 nt, accounting for 45% and 25% of the total, respectively (Figure 3B). For CP-derived t-siRNAs, the dominant sizes were 21 nt and 22 nt (Figure 3C), whereas the most dominant sizes of CP-derived vsiRNAs were 20 nt and 21 nt (Figure 3D), suggesting that DCL4 is the major producer of CP-derived t-siRNA and that RSV vsiRNA production involves multiple DCL functions.

**FIGURE 4:**
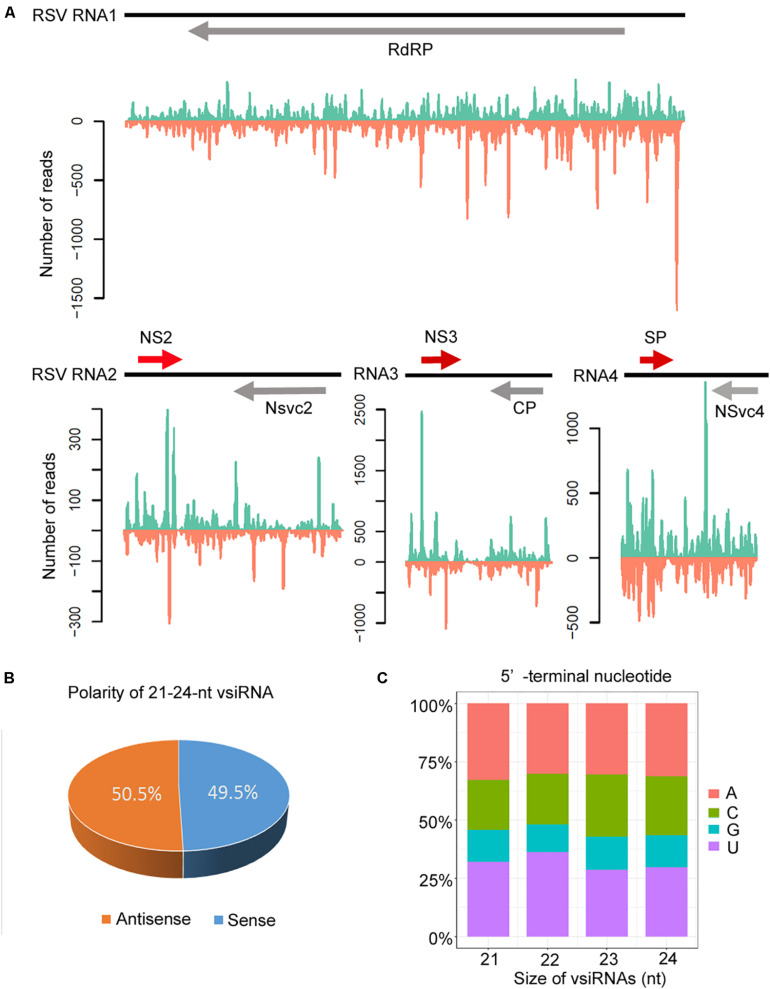
Profile of 21–24 nucleotide (nt) virus-derived small interfering (vsiRNA) derived from RSV-infected Col-0 plants. **(A)** Distribution of vsiRNAs along the RSV genome in both positive (blue) and negative (red) polarity. Genome organization of RSV positioned proportionally above. CP, coat protein; RdRp, RNA-dependent RNA polymerase; SP, disease-specific protein. **(B)** Accumulation of sense and antisense vsiRNAs. **(C)** 5′-terminal nucleotide frequency of 21–24 nt vsiRNAs.

The canonical 21–24-nt small RNAs from the viral sequences were further analyzed. The 21–24-nt vsiRNA reads were mapped to the RSV genomic sequence to explore their origin. As shown in Figure 4A and Supplementary Figures S2, S3, the proportions of vsiRNAs from both polarities were almost continuous, but some genomic regions showed higher mapping frequencies. In the RSV RNA1 genome, the complementary strand of the 3′ terminus produced significantly more vsiRNAs, and other RNA segments also exhibited some hotspots for vsiRNA generation (Figure 4A and Supplementary Figures S2, S3). The sense and antisense RSV RNA strands generated almost equivalent amounts of sense (49.5%) and antisense (50.5%) vsiRNA (Figure 4B). In addition, preferential occurrence of A/U at the 5′ terminus was identified in vsiRNAs (Figure 4C). These results suggest that RSV vsiRNAs are potentially loaded into AGO1/AGO2-containing RISCs.

### Characterization of t-siRNA in NOP-CP Transgenic *Arabidopsis* Plants

**FIGURE 5:**
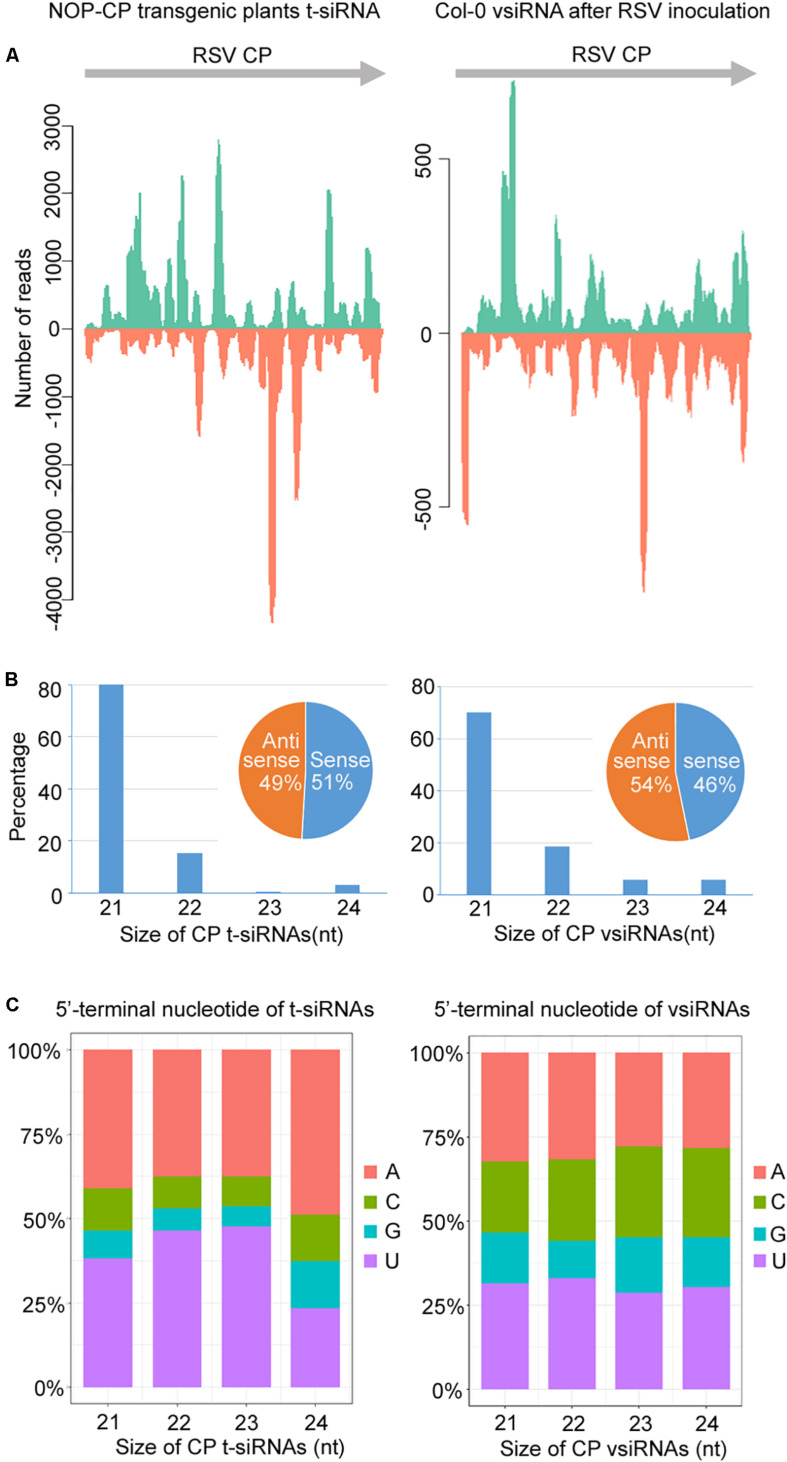
Characterization and comparison of 21–24 nt transgenic-derived small interfering RNA (t-siRNA) mapped to the CP sequence in NOP-CP transgenic plants (left) and virus-derived small interfering RNA (vsiRNA) mapped to the RSV CP sequence in RSV-infected Col-0 plants (right). **(A)** Distribution of t-siRNAs and vsiRNAs along the CP sequence in both positive (blue) and negative (red) polarity. Note that the scale used for the NOP-CP transgenic plants is different from that used for the RSV-infected Col-0 plants. **(B)** Size distribution of t-siRNAs and vsiRNAs. Pie graph showing the percentage of the sense and antisense t-siRNAs and vsiRNAs. **(C)** 5′-terminal nucleotide frequency of 21–24 nt t-siRNAs and vsiRNAs.

To understand the significance of t-siRNA in CP-mediated RSV resistance, we characterized and compared t-siRNAs generated from the NOP-CP transgene of transgenic *Arabidopsis* plants and vsiRNAs mapping to the same region (CP-derived vsiRNA) in RSV-infected wild-type plants. The sequence distribution profile of 21–24-nt t-siRNA from the NOP-CP transgene region showed that t-siRNA accumulated in several hotspot regions throughout the transgenic sequence. Interestingly, CP vsiRNAs mapping to the CP region also showed a very similar profile, with peaks in the same position, although the t-siRNA peak read number was much higher than that for vsiRNAs (Figure 5A and Supplementary Figure S4). The similar t-siRNA and vsiRNA distribution profiles indicated that a sequence bias in the t-siRNA population depends on a nucleotide sequence preference and that there is a similar mechanism for t-siRNA and vsiRNA generation.

The size distributions of t-siRNAs showed that 21 nt was the most dominant size, representing 80% of the total t-siRNAs (Figure 5B). Similarly, the 21-nt classes accounted for the majority of the CP vsiRNAs, representing 70% of the total CP vsiRNAs (Figure 5B), indicating similar sRNA processing properties in transgene- and virus-derived sRNA. To explore the origin of t-siRNAs, the strand specificity and locations of t-siRNAs were analyzed. As shown in Figures 5A,B, almost equivalent proportions of sense and antisense sRNAs were generated in both t-siRNAs and CP vsiRNAs, although the sense (51%) t-siRNA amounts were slightly greater than the antisense orientation amounts (49%), whereas for CP vsiRNAs, more reads were counted in the antisense (54%) orientation. The 5′ terminal nucleotides were characterized for 21–24 nt t-siRNAs and CP-vsiRNAs. The U/A were the most abundant 5′ nucleotides in all four sizes of both t-siRNAs and CP-vsiRNAs (Figure 5C).

Furthermore, we also characterized and compared the t-siRNA populations of NOP-CP transgenic *Arabidopsis* plants with mock treatment and RSV inoculation. The results showed that the t-siRNAs of NOP-CP transgenic plants after mock and RSV inoculation shared highly similar distribution profiles, although the read number of total t-siRNAs in RSV inoculation plants was significantly less than that in the mock treatment plants (Supplementary Figure S5 and Table 1). Characterization of t-siRNAs in NOP-CP transgenic *Arabidopsis* plants indicates that NOP-CP-derived t-siRNAs may be associated with CP-mediated RSV resistance in *Arabidopsis*.

### Plant DCLs 2, 3, and 4 Are Indispensable for CP-Mediated RSV Resistance in *Arabidopsis*

**FIGURE 6:**
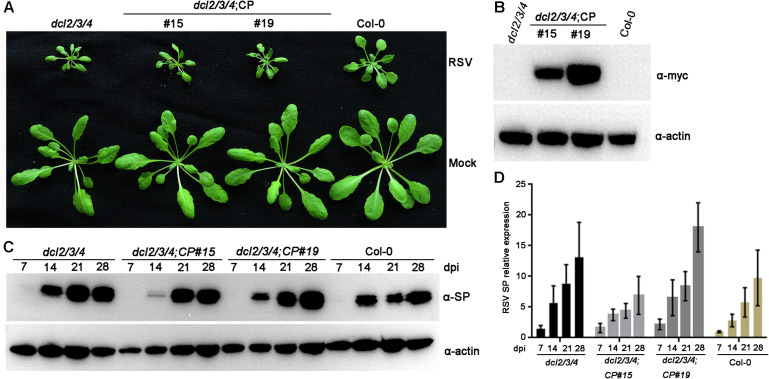
Rice stripe virus resistance examination of CP transgenic plants in the *dcl2/3/4* background. **(A)** Symptoms of mock-inoculated and RSV-infected CP transgenic plants in the *dcl2/3/4* background (*dcl2/3/4*;CP#15, *dcl2/3/4*;CP#19) and *dcl2/3/4* and Col-0 *Arabidopsis* plants. Photographs were taken at 4 weeks post-inoculation. **(B)** Western blot analysis of CP transgenic lines in the *dcl2/3/4* background with an anti-Myc antibody. The actin protein level served as a loading control. **(C)** Western blot analysis of the time course of RSV-encoded SP protein accumulation in RSV-infected CP transgenic plants in the *dcl2/3/4* background and *dcl2/3/4* (*dcl2/3/4*;CP#15, *dcl2/3/4*;CP#19) and Col-0 *Arabidopsis* plants using an SP-specific antibody. The actin protein level served as a loading control. **(D)** qRT-PCR analysis for the time course of RSV SP mRNA transcription levels in RSV-infected CP transgenic plants in the *dcl2/3/4* background (*dcl2/3/4*;CP#15, *dcl2/3/4*;CP#19) and *dcl2/3/4* and Col-0 *Arabidopsis* plants. Signal intensities for each transcript were normalized with those for *EF1-α*. Values are means ± SD (*n* = 3).

Previous studies have demonstrated that plant DCLs are responsible for generating transgene-derived t-siRNAs and virus-derived vsiRNAs (Guo et al., 2019). To test our hypothesis that the accumulation of t-siRNAs is associated with RSV resistance in CP transgenic *Arabidopsis* plants, we generated CP transgene plants in the *Arabidopsis dcl2/3/4* mutant background. These T2 generation transgenic plants were confirmed by western blot using an anti-Myc antibody (Figure 6B). An RSV infection assay showed that CP transgenic *Arabidopsis* plants in the *dcl2/3/4* background (*dcl2/3/4*;CP) developed severe stunting symptoms similar to those of non-transgenic *dcl2/3/4* and Col-0 plants (Figure 6A). Western blot and qRT-PCR results both showed that both RSV SP mRNA and proteins accumulated to similar levels in *dcl2/3/4*;CP plants and non-transgenic *dcl2/3/4* and Col-0 plants (Figures 6C,D and Supplementary Figure S6). These results indicate that CP-mediated RSV resistance depends on the function of DCLs in *Arabidopsis*.

## Discussion

Since the concept of pathogen-derived resistance (PDR) was proposed (Sanford and Johnston, 1985) and confirmed by expressing tobacco mosaic virus (TMV) CP in transgenic tobacco plants, resulting in TMV resistance (Abel et al., 1986), CP-mediated resistance has been widely used to protect various plant species against virus infection (Galvez et al., 2014). In the case of RSV, the overexpression of CP in rice plants resulted in partial resistance to virus infection, but no related mechanism was determined (Hayakawa et al., 1992). Recent studies have also demonstrated that the RSV CP triggers jasmonate accumulation and subsequently induces the plant antiviral immune response (Han et al., 2020; Yang et al., 2020) and that RSV CP-mediated resistance requires AGO18 protein expression in rice plants (Yang et al., 2020). Thus, RSV CP-mediated resistance appears to be a protein-mediated phenomenon in rice plants, similar to TMV CP-mediated resistance in tobacco plants (Lindbo and Falk, 2017). However, the effectiveness of transgene-induced silencing could not be ruled out in these previous studies, as some resistant lines containing translatable transgenes also generated RNA-mediated resistance correlating with t-siRNAs. In this study, we provide evidence that the expression of RSV CP in transgenic *Arabidopsis* plants is not a prerequisite for CP-mediated resistance. First, both translatable and non-translatable versions of CP transgenic *Arabidopsis* plants all showed immunity to RSV infection. Second, by using deep sequencing analysis, CP-mediated resistance in transgenic *Arabidopsis* plants was associated with high accumulation of 21–24-nt t-siRNAs. Third, CP overexpression in the *dcl2/3/4* mutant background resulted in similar sensitivity to RSV infection in non-transgenic *dcl2/3/4* plants. Our results strongly indicate that RSV CP-mediated resistance is due to an RNA silencing-mediated resistance mechanism in *Arabidopsis*, similar to that indicated in tobacco etch virus (TEV) CP-mediated resistance (Lindbo et al., 1993) and tomato spotted wilt virus (TSWV) N (nucleocapsid)-mediated resistance (Catoni et al., 2013).

RNA silencing-directed resistance is mediated by 21–24 nt siRNAs (Hamilton et al., 2002; Ruiz-Ferrer and Voinnet, 2009) generated through the cleavage of a double-stranded or an imperfect stem-loop RNA molecule by plant DCLs (Moazed, 2009). In this paper, Illumina deep sequencing was performed to characterize the small RNA population and gain insights into the RNA-based virus resistance mechanism during RSV-*Arabidopsis* interactions. Bioinformatic analysis of the deep sequencing data indicated that RSV infection triggered the generation of relatively large amounts of vsiRNA, accounting for 1.55% of the total 18–30-nt reads in *Arabidopsis* plants (Table 1), in contrast with an average of 0.29% of the total small RNA reads mapped to the RSV genome in RSV-infected natural host rice plants (Yang et al., 2018). This difference is probably a result of the different virus titers in different RSV-host pathosystems. In general, DCL4-generated 21-nt vsiRNAs are the most abundant class, following DCL-dependent 22-nt vsiRNAs (Guo et al., 2019). In RSV-infected *Arabidopsis*, 20-nt vsiRNAs was the most abundant species (Figure 3B). This result is similar to the rice ragged stunt virus (RRSV) vsiRNAs in rice plants (Li et al., 2018). In RSV-infected rice and tobacco plants, 20 nt vsiRNAs also account for the high proportion (Yan et al., 2010; Jiang et al., 2012; Xu et al., 2012). These results indicate that 20 nt vsiRNAs may play a role in RSV-plants interaction. The mechanism of biogenesis and function of 20 nt visRNA remains elusive, though the biogenesis of 20 nt miRNAs is dependent on DCL1 (Lee et al., 2015). Further studies are needed to elucidate the role of *Arabidopsis* DCL1 in the production RSV vsiRNAs.

In *Arabidopsis*, AGO1 and AGO2 play crucial roles in antivirus defense (Carbonell and Carrington, 2015) and associate with small RNAs that exhibit uridine and adenosine at their 5′ ends, respectively (Mi et al., 2008). Our study also revealed preferential occurrence of uridine or adenosine residues at the 5′ terminus in RSV vsiRNA, showing the conserved AGO complexes binding vsiRNA for antivirus defense. Compared with previous studies, the 5′-terminal nucleotides of RSV visRNAs from *Arabidopsis*, rice and tobacco plants were similar (Yan et al., 2010; Jiang et al., 2012; Xu et al., 2012; Yang et al., 2018), suggesting that AGO1 and AGO2 play an important role in virus defense against RSV in plants.

Regarding the genomic regions from which RSV vsiRNAs were generated, fewer vsiRNAs were produced from RNA2 than from the other three RNA segments in *Arabidopsis* (Figure 4); however, in RSV-infected rice plants, RNA1 generated fewer vsiRNAs than the other three RNA segments (Xu et al., 2012; Yang et al., 2018). These distinct RSV vsiRNA origins in plant hosts might be explained by the evolutionary adaptation of RSV, which escapes RNA silencing antivirus machinery, thus facilitating successful virus infection in rice plants. In this study, RSV-derived vsiRNAs equally originate from the sense and antisense strands (Figure 4B), consistent with previous reports in RSV-rice interaction (Yan et al., 2010; Yang et al., 2018). These results indicate that the RSV vsiRNAs may originated from double-stranded replication intermediates.

To characterize CP t-siRNAs that confer resistance to RSV infection, the origin, composition and abundance of CP sequence-derived t-siRNAs were analyzed and compared with those of CP-derived vsiRNAs. The sequence distribution, 5′ terminal nucleotides and strand specificity exhibited very similar patterns between CP-derived t-siRNAs and CP-derived vsiRNAs, except that the amount of t-siRNAs was nearly twice that of vsiRNAs, indicating that the activation of t-siRNAs mediates antiviral defense derived from the transgene. A prerequisite step for t-siRNA biogenesis is a sufficient quantity of transgene RNA that has a dsRNA nature. Multiple studies have demonstrated that transgenes can often integrate into the host genome in complex structures, such as multiple copies inverted to each other, and transcription of transgenic DNA from these complex integrations could lead to dsRNA structures (Lindbo and Falk, 2017). Another possibility is that the single-stranded RNA transcript from transgene DNA is converted to dsRNAs by SUPPRESSOR OF GENE SILENCING 3 (SGS3) and RNA-DEPENDENT RNA POLYMERASE 6 (RDR6) (Guo et al., 2019). Further genetic and biochemical studies will be necessary to address which pathway is responsible for t-siRNA biogenesis in CP transgenic *Arabidopsis* plants.

In *Arabidopsis* plants, DCL4, DCL2 and DCL3 process transgene-derived dsRNAs to 21–24 nt siRNAs in a redundant and hierarchical manner (Deleris et al., 2006), which are incorporated into AGO1 or AGO2 effectors to guide cleavage of target RNAs (Pumplin and Voinnet, 2013). To further confirm our hypothesis that the accumulation of t-siRNAs was associated with RSV immunity in CP transgenic *Arabidopsis* plants, CP transgenic plants in the *dcl2/3/4* triple mutant background were developed. As expected, these transgenic plants exhibited susceptibility to RSV infection similar to that of non-transgenic plants, thus supporting our hypothesis. Our results also showed that *dcl2/3/4* triple mutant plants exhibited increased severity of disease symptoms and virus titers compared with those of Col-0 plants in RSV challenge assays, and similar results were obtained with other RNA viruses (Guo et al., 2019). Further investigation is needed to elucidate the special role of DCLs in producing t-siRNAs in CP transgenic *Arabidopsis* plants. The mechanism of RSV CP-mediated virus resistance revealed by this study provides an in-depth understanding of RNA-based antiviral immunity in RSV-*Arabidopsis* interactions.

## Data Availability Statement

The datasets presented in this study can be found in online repositories. The names of the repository/repositories and accession number(s) can be found in the article/Supplementary Material.

## Author Contributions

FS and TZ conceived and designed the experiments. FS, PH, and WW conducted the experiments. YL, LD, FS, and TZ analyzed the data. FS and TZ wrote the manuscript. All authors read and approved the manuscript.

## Conflict of Interest

The authors declare that the research was conducted in the absence of any commercial or financial relationships that could be construed as a potential conflict of interest.

## Supplementary Material

Supplementary Figure S1 Characterization and expression of CP-YFP and NOP-CP-YFP in *Nicotiana benthamiana*. **(A)** Confocal imaging assays show the expression of CP-YFP but not NOP-CP-YFP in *Nicotiana benthamiana*. **(B)** RT-PCR analysis of CP mRNA transcription levels of CP-YFP and NOP-CP-YFP in *Nicotiana benthamiana.* The *ubc* mRNA level served as a loading control. **(C)** Western blot analysis of the expression of CP-YFP and NOP-CP-YFP in *Nicotiana benthamiana* using an YFP-specific antibody. The actin protein level served as a loading control.

Supplementary Figure S2 Profile of 21–24 nucleotide (nt) virus-derived small interfering RNA (vsiRNA) derived from RSV-infected Col-0 plants, biological replicate 2.

Supplementary Figure S3 Profile of 21–24 nucleotide (nt) virus-derived small interfering (vsiRNA) derived from RSV-infected Col-0 plants biological replicate 3.

Supplementary Figure S4 Characterization and comparison of 21–24 nt transgenic-derived small interfering RNA (t-siRNA) mapped to the CP sequence in NOP-CP transgenic plants **(left)** and virus-derived small interfering RNA (vsiRNA) mapped to the RSV CP sequence in RSV-infected Col-0 plants **(right)** from two other biological replicates.

Supplementary Figure S5 Characterization and comparison of 21–24 nt transgenic-derived small interfering RNA (t-siRNA) mapped to the CP sequence in NOP-CP transgenic plants after mock control (left) and RSV inoculation (right). **(A)** Distribution of t-siRNAs along the CP sequence in both positive (blue) and negative (red) polarity. Note that the scale used for the mock control is different from that used for the RSV inoculation. **(B)** Size distribution of t-siRNAs. Pie graph showing the percentage of the sense and antisense t-siRNAs. **(C)** 5′-terminal nucleotide frequency of 21–24 nt t-siRNAs.

Supplementary Figure S6 Comparison of RSV resistance in CP transgenic plants in *dcl2/3/4* and Col-0 background. **(A)** Western blot analysis of RSV-encoded SP protein accumulation in RSV-infected CP transgenic *Arabidopsis* plants in *dcl2/3/4* and Col-0 background at 28 dpi. The actin protein level served as a loading control. **(B)** qRT-PCR analysis of RSV SP mRNA transcription levels in RSV-infected CP transgenic plants in *dcl2/3/4* and Col-0 background at 28 dpi. Signal intensities for each transcript were normalized with those for *EF1-α*. Values are means ± SD (*n* = 3). ^∗^*p* ≤ 0.05 (Student’s *t*-test).

Supplementary Table S1 Primer sequence used for the cloning of transgenes and testing the viral infection.
