# Supplementary figures and images for: Rice Stripe Virus Coat Protein-Mediated Virus Resistance Is Associated With RNA Silencing in Arabidopsis
Source: Front Microbiol. 2020 Nov 13;11:591619. doi: 10.3389/fmicb.2020.591619 (PMC7691420; doi:10.3389/fmicb.2020.591619)

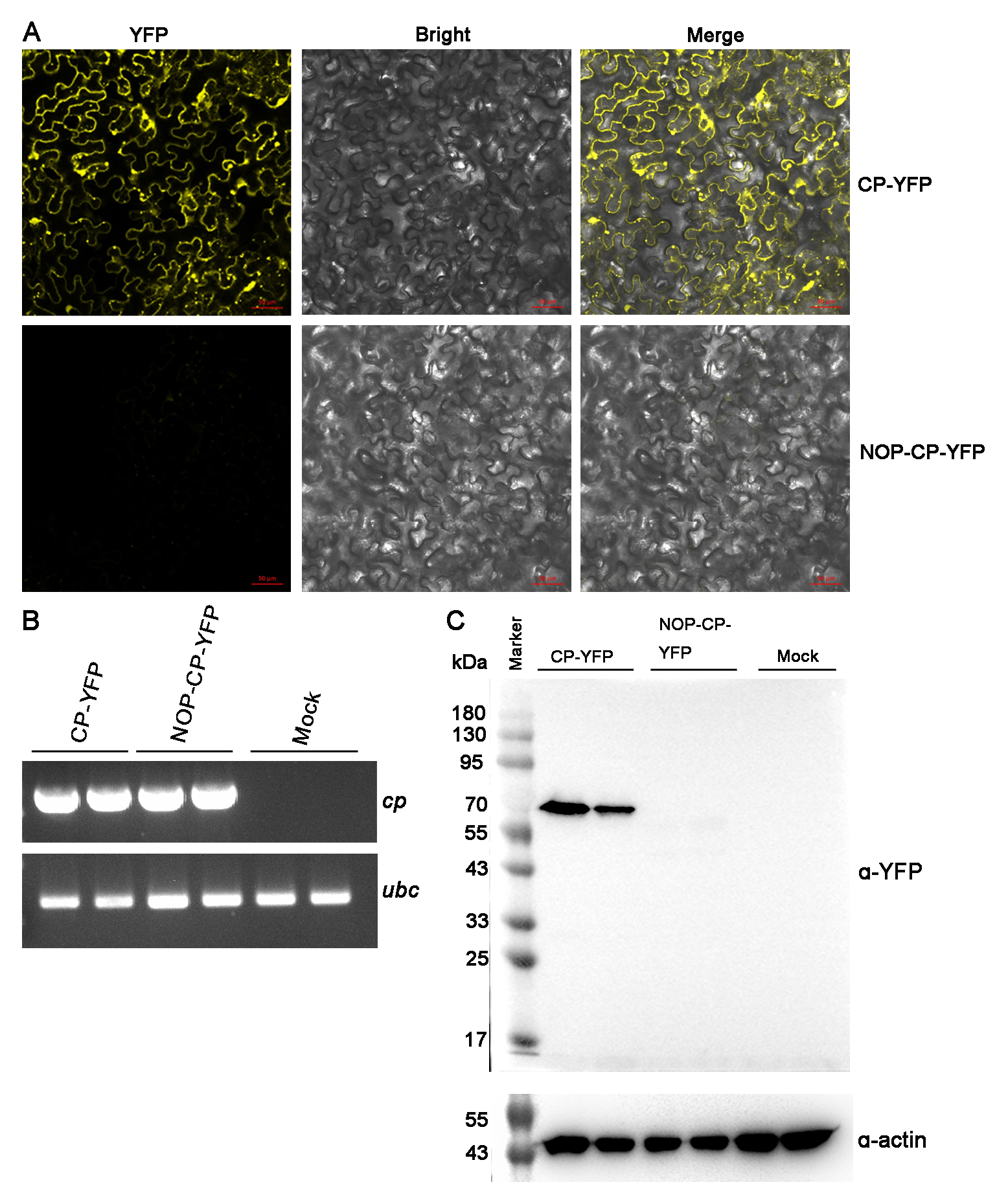

Supplement: Supplementary Figure S1 — Characterization and expression of CP-YFP and NOP-CP-YFP in Nicotiana benthamiana. (A) Confocal imaging assays show the expression of CP-YFP but not NOP-CP-YFP in Nicotiana benthamiana. (B) RT-PCR analysis of CP mRNA transcription levels of CP-YFP and NOP-CP-YFP in Nicotiana benthamiana. The ubc mRNA level served as a loading control. (C) Western blot analysis of the expression of CP-YFP and NOP-CP-YFP in Nicotiana benthamiana using an YFP-specific antibody. The actin protein level served as a loading control. [file Image_1.TIF]

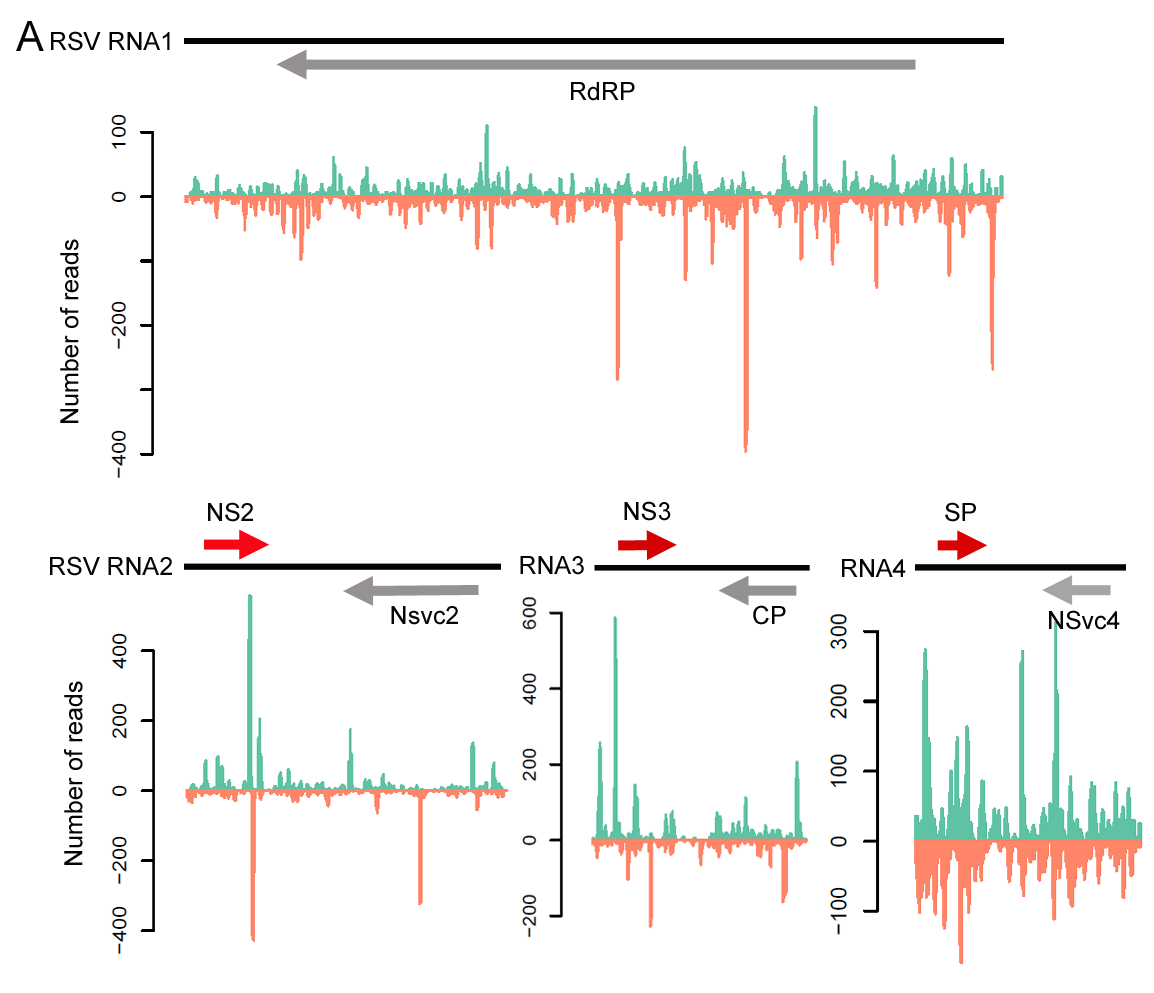

Supplement: Supplementary Figure S2 — Profile of 21–24 nucleotide (nt) virus-derived small interfering RNA (vsiRNA) derived from RSV-infected Col-0 plants, biological replicate 2. [file Image_2.TIF]

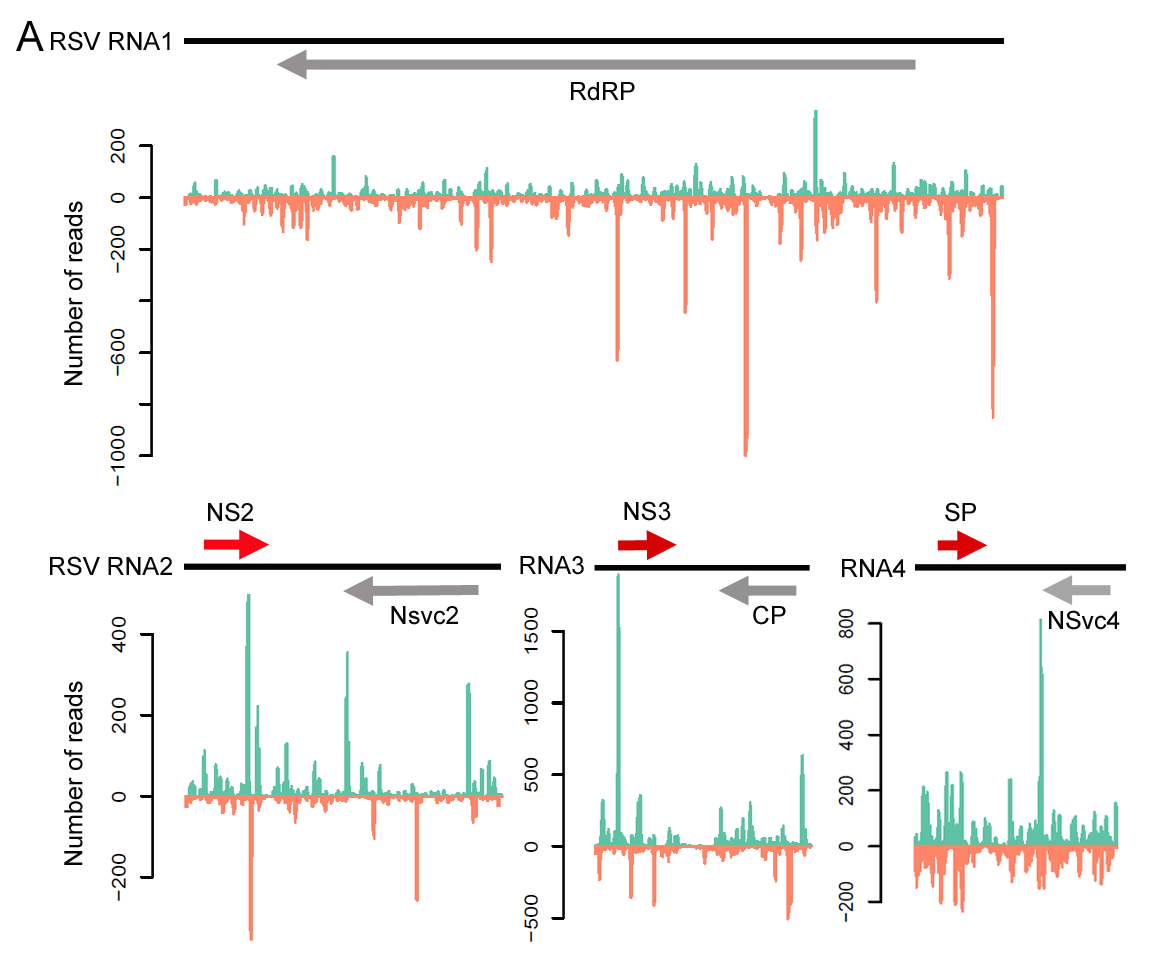

Supplement: Supplementary Figure S3 — Profile of 21–24 nucleotide (nt) virus-derived small interfering (vsiRNA) derived from RSV-infected Col-0 plants biological replicate 3. [file Image_3.TIF]

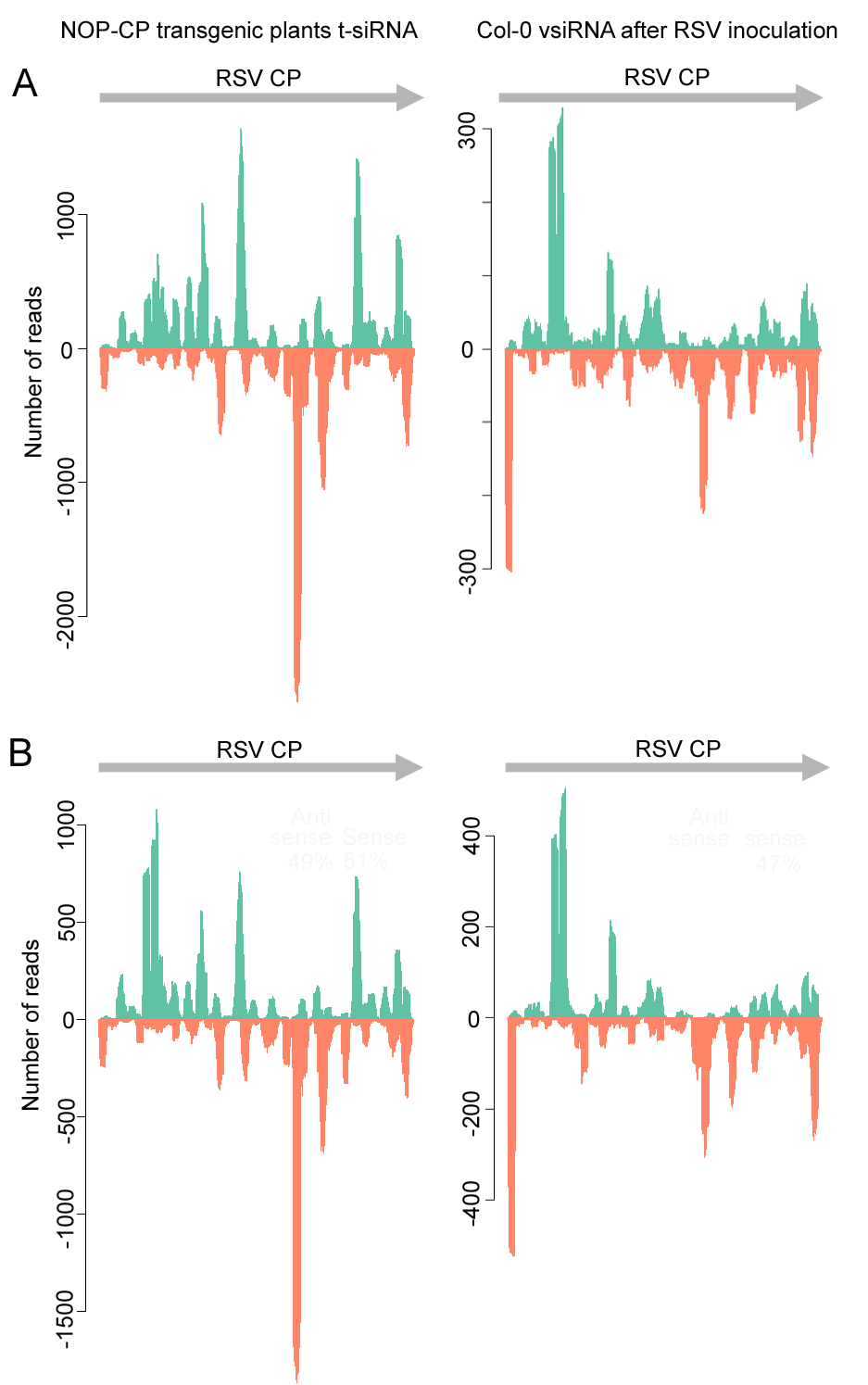

Supplement: Supplementary Figure S4 — Characterization and comparison of 21–24 nt transgenic-derived small interfering RNA (t-siRNA) mapped to the CP sequence in NOP-CP transgenic plants (left) and virus-derived small interfering RNA (vsiRNA) mapped to the RSV CP sequence in RSV-infected Col-0 plants (right) from two other biological replicates. [file Image_4.TIF]

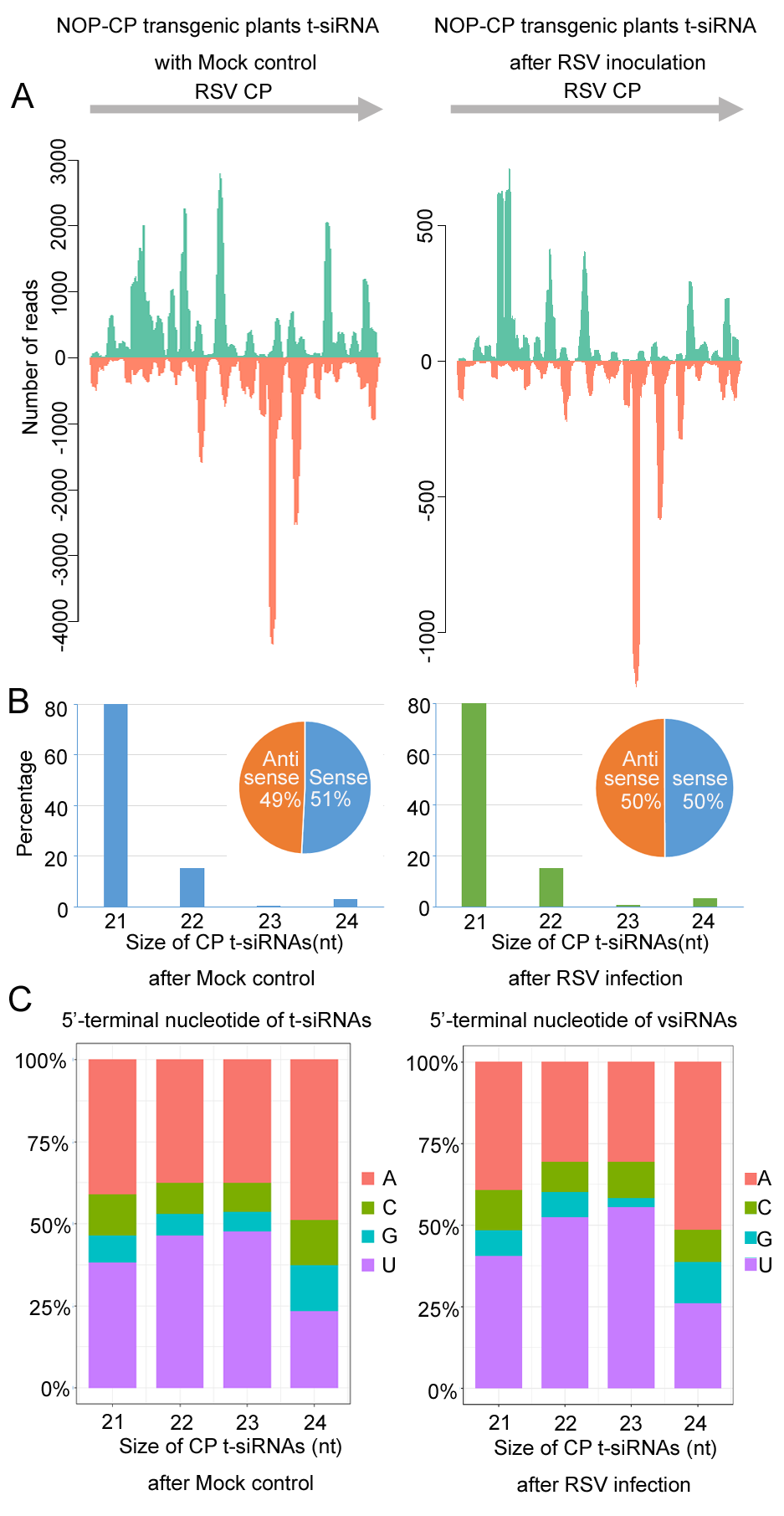

Supplement: Supplementary Figure S5 — Characterization and comparison of 21–24 nt transgenic-derived small interfering RNA (t-siRNA) mapped to the CP sequence in NOP-CP transgenic plants after mock control (left) and RSV inoculation (right). (A) Distribution of t-siRNAs along the CP sequence in both positive (blue) and negative (red) polarity. Note that the scale used for the mock control is different from that used for the RSV inoculation. (B) Size distribution of t-siRNAs. Pie graph showing the percentage of the sense and antisense t-siRNAs. (C) 5′-terminal nucleotide frequency of 21–24 nt t-siRNAs. [file Image_5.TIF]

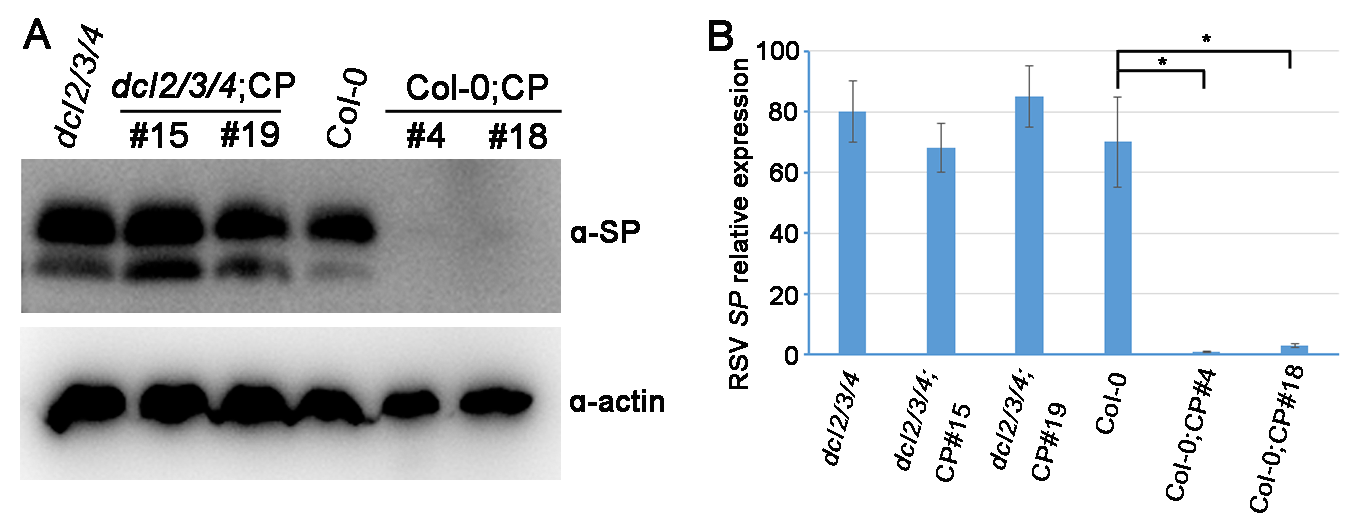

Supplement: Supplementary Figure S6 — Comparison of RSV resistance in CP transgenic plants in dcl2/3/4 and Col-0 background. (A) Western blot analysis of RSV-encoded SP protein accumulation in RSV-infected CP transgenic Arabidopsis plants in dcl2/3/4 and Col-0 background at 28 dpi. The actin protein level served as a loading control. (B) qRT-PCR analysis of RSV SP mRNA transcription levels in RSV-infected CP transgenic plants in dcl2/3/4 and Col-0 background at 28 dpi. Signal intensities for each transcript were normalized with those for EF1-α. Values are means ± SD (n = 3). ∗p ≤ 0.05 (Student’s t-test). [file Image_6.TIF]
