# Supplementary material for: Rice Stripe Virus Coat Protein-Mediated Virus Resistance Is Associated With RNA Silencing in Arabidopsis
Source: Front Microbiol. 2020 Nov 13;11:591619. doi: 10.3389/fmicb.2020.591619 (PMC7691420; doi:10.3389/fmicb.2020.591619)
Supplement: Supplementary Table S1 — Primer sequence used for the cloning of transgenes and testing the viral infection. [file Table_1.docx]

Table S1. Primers sequences used for cloning of transgenes and testing the viral infection.

| Gene | Primers | Product Size (bp) | tests |
| --- | --- | --- | --- |
| *CP* | 5’- GGGGACAAGTTTGTACAAAAAAGCAGG CTTCATGGGTACCAACAAGCCAGCCACT -3’  5’- GGGGACCACTTTGTACAAGAAAGCTGGGT CCTAGTCATCTGCACCTTCTGCCTCG -3’ | 960 | clone |
| *NOP-CP* | 5’- GGGGACAAGTTTGTACAAAAAAGCAGG CTTCTAGGGTACCAACAAGCCAGCCACT -3’  5’- GGGGACCACTTTGTACAAGAAAGCTGGGT CCTAGTCATCTGCACCTTCTGCCTCG -3’ | 960 | clone |
| *CP* | 5’-AGGATGTGACAACTTACTGTGGGACT-3’  5’-GACTTGCATGTGATGACCAGGAGA-3’ | 237 | RT-PCR |
| *SP* | 5’-TTGTCACTCATTCTTATCACACCTG-3’  5’-TTCTTCCACACTTTCTCATACTCTT-3’ | 237 | qRT-PCR |
| *EF1-a* | 5’-GGCTGCTGAGATGAACAA-3’  5’-GTGGTGGAGTCAATGATAAG-3’ | 225 | qRT-PCR  RT-PCR |
| *Ubc* | 5’-GAGGAAGAGACTGGTGAGGGAT-3’  5’-CACAGAGCAAAGACTGGATTGA-3’ | 329 | RT-PCR |
